# Editorial: Lactation genomics and phenomics in farm animals: Where are we at?

**DOI:** 10.3389/fgene.2023.1173595

**Published:** 2023-04-18

**Authors:** Xiao-Lin Wu, Xiangdong Ding, Yunxia Zhao, Asha M. Miles, Luiz F. Brito, Bjorg Heringstad, Shuhong Zhao, Zhihua Jiang

**Affiliations:** ^1^ Council on Dairy Cattle Breeding, Bowie, MD, United States; ^2^ Department of Animal Sciences, University of Wisconsin, Madison, WI, United States; ^3^ College of Animal Science and Technology, China Agricultural University, Beijing, China; ^4^ College of Animal Science and Technology, Huazhong Agricultural University, Wuhan, China; ^5^ Animal Genomics and Improvement Laboratory, USDA, Agricultural Research Service, Beltsville, MD, United States; ^6^ Department of Animal Sciences, Purdue University, West Lafayette, IN, United States; ^7^ Department of Animal and Aquacultural Sciences, Faculty of Biosciences, Norwegian University of Life Sciences, Aas, Norway; ^8^ Department of Animal Science, Washington State University, Pullman, WA, United States

**Keywords:** milk, genomic prediction, GWAS, high-throughput phenotyping, SNP

Lactation is a crucial process for dairy animals, as it provides the primary source of nutrition for their offspring, and a balanced source of nutrients for human consumption. The studies of lactation genomics involve investigating the genome’s structure, function, evolution, and regulation that underly lactation biology. Over the past two decades, genomics has revolutionized dairy cattle breeding worldwide, leading to significantly reduced generation intervals and increased genetic gain by year (Wiggans et al., 2017). Dairy farmers can now use genomic bulls for more accurate selection instead of waiting for progeny testing results. However, the success of genomic selection depends on comprehensive phenotyping, i.e., phenomics. The latter involves generating high-dimensional and close-to-biology phenotypic data on an animal-wide scale (Houle et al., 2010). This approach breaks down composite traits into more direct indicators of ultimate breeding goals that can be easily measured in large-scale farming applications (Brito et al., 2020). Livestock breeders have used complex selection indices to combine many traits into a single performance measurement for decades. Now, there is a renewed interest in collecting high-throughput data on individual animals driven by various research initiatives and promising technologies for massive, low-cost, and accurate phenotypes (Cole et al., 2020). The latest 10-year blueprint for animal genomics research, led by the U.S. Department of Agriculture, emphasizes the need to close the genome-to-phenome gaps (Rexroad et al., 2019).

This Research Topic comprises nine papers on a wide range of topics related to lactation genomics and phenomics. The accuracy of milk records is fundamental to farm management decisions and genomic predictions. Since the 1960s, cost-effective milk recording routines have been adapted to supplement the standard supervised twice-daily monthly testing scheme, assuming equal morning (AM) and evening (PM) milking intervals. However, in reality, the AM and PM milking intervals can vary considerably. Wu et al. characterized and compared additive (ACF) and multiplicative (MCF) correction factors. ACFs provide additive adjustments beyond twice AM or PM yields, while MCFs represent daily to partial milk yield ratios, although their mathematical forms and statistical interpretations vary. Overall, the MCF and linear regression models outperformed the ACF models. An exponential regression model was proposed, analogous to an exponential growth function with the yield from single milking as the initial state and the rate of change tuned by a linear function of milking interval (Wu et al., 2022). This model provided the most accurate estimates of test-day milk yields. However, discretizing milking intervals into large categories was a major concern because it led to substantial accuracy loss, regardless of the model used.

Genome-wide association studies (GWAS) and differential gene expression profiling remain the main tools for discovering genes that determine and regulate milk production and other relevant traits. Wang et al. identified seven significant SNPs associated with multiple traits that link to candidate genes with known functionalities in fat metabolism or mammary gland development. Pan et al. estimated genetic parameters and reported associations for milk production traits and somatic cell scores that varied across different lactation stages of the Shanghai Holstein population. Lin et al. showed that the long-chain acyl-CoA synthetase 1 (ACSL1) gene, which plays a vital role in fatty acids metabolism and is highly expressed in the lactating mammary gland epithelial cells of lactating animals, was associated with milk production performance in buffalos. Through a comparative analysis of expression patterns in non-lactation and lactation mammary glands of goats, sheep, and cows, Zhang et al. postulated that two ACS genes, ACSS2 and ACSF3, could participate in the formation mechanisms of the goat milk flavor. Using differential gene expression analysis in the mammary glands, Sadovnikova et al. reported a direct relationship between the response to dexamethasone, an exogenous glucocorticoid, and the concurrent suppression of milk yield due to the reduced synthesis of α -lactalbumin and lactose by the mammary epithelium. Farhadian et al. reported potential lactation- and breed-specific SNPs in the regions of QTL and candidate genes associated with milk production using a transcriptome approach.

Genomic selection in indigenous breeds or minor breeds is often limited by size of available training populations. Therefore, combining phenotypic, pedigree, and genomic data from genetically related populations can be a feasible strategy to overcome this limitation. Teissier et al. evaluated the genetic connectedness and population structure of dairy goats from four countries and found that international genomic evaluations are feasible, especially for French and Italian goats. Using whole-genome sequence (WGS) data can enhance understanding of the genomic background of economic traits such as milk production in farm animals and the genomic predictions of genomic breeding values (Meuwissen et al., 2021). Jiang et al. demonstrated that genotype imputation from SNP arrays to WGS data was a cost-effective approach to obtain high-density genotypes for GWAS and genomic predictions, subject to model parameter optimization.

Still, the coverage of the nine topics on lactation genomics and phenomics is very limited. Appealing subjects yet not addressed include dairy breeding focusing on adaptation and environmental resilience, genomics solutions to metabolic and nutritional problems related to milk production, genomic mating toward sustainable dairy breeding, and integration of multi-omics to understand the biological mechanisms underlying lactation physiology better, to name a few. In advanced countries, high-throughput phenotyping is a reality in dairy farming. For example, large dairy farms or operations have adopted automatic milking systems capable of massive recording of phenotypes (Pedrosa et al., 2023). Precision livestock farming tools are being developed and used to collect detailed, in-depth, and high-through measurements about animal productivity, health, environmental efficiency, and welfare and their environments in or near real-time. However, these new phenotyping technologies are proprietary when offered to dairy producers, and they lack independent and unbiased validation (Cole et al., 2020). Developing non-invasive techniques for measuring phenotypic traits can improve animal welfare and reduce the costs and time associated with phenotyping, such as 3D imaging and infrared spectroscopy. New phenotypes are emerging, though their roles in genetic improvement programs remain unclear. Looking forward, we expect that integrating lactation phenomics and genomics will continue to provide a more comprehensive understanding of lactation biology and aid in developing better tools for dairy management and genetic improvement.

## References

[B1] Brito L. F. Oliveira H. R. McConn B. R. Schinckel A. P. Arrazola A. Marchant-Forde J. N. (2020). Large-scale phenotyping of livestock welfare in commercial production systems: A new frontier in animal breeding. Front. Genet. 11, 793. 10.3389/fgene.2020.00793 32849798 PMC7411239

[B2] Cole J. B. Eaglen S. A. E. Maltecca C. Mulder H. A. Pryce J. E. (2020). The future of phenomics in dairy cattle breeding. Anim. Front. 10 (2), 37–44. 10.1093/af/vfaa007 32257602 PMC7111603

[B4] Houle D. Govindaraju D. R. Omholt S. (2010). Phenomics: The next challenge. Nat. Rev. Genet. 11, 855–866. 10.1038/nrg2897 21085204

[B5] Meuwissen T. van den Berg I. Goddard M. (2021). On the use of whole-genome sequence data for across-breed genomic prediction and fine-scale mapping of QTL. Genet. Sel. Evol. 53, 19. 10.1186/s12711-021-00607-4 33637049 PMC7908738

[B6] Pedrosa V. B. Boerman J. P. Gloria L. S. Chen S. Y. Montes M. E. Doucette J. S. (2023). Genomic-based genetic parameters for milkability traits derived from automatic milking systems in North American Holstein cattle. J. Dairy Sci. 106, 2613–2629. 10.3168/jds.2022-22515 36797177

[B7] Rexroad R. Vallet J. Matukumalli L. K. Reecy J. Bickhart D. Blackburn H. (2019). Genome to phenome: Improving animal health, production, and well-being – a new USDA blueprint for animal genome research 2018–2027. Front. Genet. 10, 327. 10.3389/fgene.2019.00327 31156693 PMC6532451

[B8] Wiggans G. R. Cole J. B. Hubbard S. M. Sonstegard T. S. (2017). Genomic selection in dairy cattle: The USDA experience. Annu. Rev. Anim. Biosci. 5, 309–327. 10.1146/annurev-animal-021815-111422 27860491

[B9] Wu X.-L. Wiggans G. R. Norman H. D. Miles A. M. Van Tassell C. P. Baldwin VI R. L. (2022). Daily milk yield correction factors: What are they? JDS Commun. 4 (1), 40–45. 10.3168/jdsc.2022-0230 36713119 PMC9873820

